# Short-term safety and efficacy of Preserflo™ Microshunt in glaucoma patients: a multicentre retrospective cohort study

**DOI:** 10.1038/s41433-022-01995-7

**Published:** 2022-03-12

**Authors:** Raj Bhayani, Jose Maria Martínez de la Casa, Michele Figus, Karsten Klabe, Alessandro Rabiolo, Karl Mercieca

**Affiliations:** 1 Manchester Royal Eye Hospital, Manchester University NHS Foundation Trust, Manchester, UK; 2 Ophthalmology Unit, Department of Ophthalmology and ORL, Faculty of Medicine, Hospital Clinico San-Carlos, Universidad Complutense de Madrid, Instituto de Investigacion Sanitaria del Hospital Clinico San-Carlos (IdISSC), Madrid, Spain; 3 Department of Surgical, Medical and Molecular Pathology and Critical Care Medicine, University of Pisa, Pisa, Italy; 4 Breyer-Kaymak-Klabe Augenchirurgie, Duesseldorf, Germany; 5 Department of Ophthalmology, Gloucestershire Hospitals NHS Foundation Trust, Cheltenham, UK; 6 University Hospitals Eye Clinic, Ernst-Abbe Strasse 2, Bonn, Germany; 7 University of Manchester, Manchester, UK

**Keywords:** Outcomes research, Optic nerve diseases

## Abstract

**Background/aims:**

To evaluate 1-year success rates and safety profile of Preserflo™ Microshunt in glaucoma patients.

**Methods:**

Retrospective multicentre cohort study of 100 consecutive eyes (91 patients) from four tertiary-referral glaucoma centres. Four intraocular pressure (IOP) criteria were defined: A: IOP ≤ 21 mmHg+IOP reduction ≥20% from baseline; B: IOP ≤ 18 mmHg+IOP reduction ≥20%; C: IOP ≤ 15 mmHg+IOP reduction ≥25%; D: IOP≤12 mmHg+IOP reduction ≥30%. Success was defined as qualified or complete based on whether reached with or without medication. Primary outcome was success according to the above criteria. Secondary outcomes included: IOP, best-corrected visual acuity (BCVA), medication use, complications, postoperative interventions, and failure-associated factors.

**Results:**

Qualified and complete success rates (95% CI) at 12 months were 74%(66–83%) and 58%(49–69%) for criterion A, 72%(63–82%) and 57%(48–68%) for B, 52%(43–63%) and 47%(38–58%) for C, 29%(21–40%) and 26%(19–36%) for D. Overall median (interquartile range (IQR)) preoperative IOP decreased from 21.5(19–28) mmHg to 13(11–16) mmHg at 12 months. BCVA was not significantly different up to 12 months (*p* = 0.79). Preoperative median (IQR) number of medications decreased from 3 (2–3) to 0 (0–1) at 12 months. Twelve eyes underwent needling, five surgical revision and one device removal due to corneal oedema. There were no hypotony-related complications. Non-Caucasian ethnicity was the only risk factor consistently associated with increased failure.

**Conclusions:**

Preserflo™ Microshunt is a viable surgical option in glaucoma patients, with reasonable short-term success rates, decreased medications use, excellent safety profile, smooth postoperative care, and rapid learning curve. Success rates for the most stringent IOP cutoffs were modest, indicating that it may not be the optimal surgery when very low target IOP is required.

## Introduction

Less invasive glaucoma surgeries (LIGS), particularly sub-conjunctival drainage devices such as the Preserflo™ Microshunt (Preserflo™) (Santen, Miami, USA) and Xen-45™ (Allergan, Irvine, USA), have become more popular in treatment algorithms of progressing glaucoma patients, particularly those needing a reduction in medication burden and also those potentially more at risk of the postoperative complications attributed to conventional glaucoma surgery [1, 2]. The use of LIGS is increasing significantly compared to ‘traditional’ trabeculectomy due to the perceived risk of hypotony, intensive postoperative management and significant learning curve. A recent survey of UK glaucoma surgeons showed that the use of alternative glaucoma surgeries, including Preserflo™, has increased during the COVID-19 pandemic, mainly in an attempt to reduce postoperative visits [3].

Preserflo™ is a promising new device which potentially offers a substantial reduction in intraocular pressure (IOP) via a relatively less invasive and standardised procedure. It is composed of an inert biocompatible biomaterial called poly(styrene-block-isobutylene-block-styrene), or “SIBS,” and has been shown to have excellent biocompatibility in animal studies [4, 5]. The device is 8.5 mm long with an internal lumen of 70 µm, allowing controlled flow and maintenance of IOP above 5 mmHg, assuming normal aqueous humour production [6]. The literature about Preserflo™ is scarce. Preliminary studies have shown promising early success rates [5, 7–9] but many of these are limited by small patient numbers, short follow-up, monocentric design, and selection bias (e.g., exclusion of patients with previous ocular surgery or certain glaucoma sub-types). Understanding the advantages and limitations, and identifying optimal candidates, are important to define where this procedure should fit in glaucoma clinical management. In this multicentre study, we aimed to determine the effectiveness, safety, and risk factors for failure of Preserflo™ procedures augmented with mitomycin C (MMC) during a 12-month follow-up period in a real-world patient cohort.

## Methods

### Study design

This is an investigator-initiated, multicentre, retrospective cohort study of consecutive patients receiving a Preserflo™ with MMC augmentation between 2015 and 2019 under the clinical care of four experienced glaucoma surgeons practicing in tertiary-referral glaucoma centres across Europe. Each centre provided one-year data for the first 25 consecutive cases performed. No other specific inclusion/exclusion criteria were set to ensure high generalisability of the results. The criteria for choosing Preserflo™ were not set in advance with surgical decisions being made on an individual basis. A subset of cases from one of the surgeons were previously published as a part of a multicentre clinical study [10]. The protocol adhered to the tenets of the declaration of Helsinki and had approval from all respective study centre local ethics committees.

The following baseline preoperative variables were collected: age, eye, sex, ethnicity, best-corrected visual acuity (BCVA), IOP measurement (Goldmann applanation tonometry), number and type of glaucoma medications, glaucoma sub-type, and previous ocular procedures/surgery. The following variables were collected over follow-up: BCVA, IOP, number and type of anti-glaucoma medication, complications, postoperative needling, surgical revision and any other surgical intervention. The frequency and number of visits were decided on an individual basis by the surgeon treating the patients. The following time points were established to amalgamate heterogeneous data from the various centres: Postoperative day 1, week 1, month 1, month 6, and month 12. For each time point, we used the following intervals: postoperative day 1: 1–3 days, week 1: 4–14 days, month 1: 15–59 days, month 6: 121–270 days and month 12: 271–455 days. These intervals were chosen based on the recommendations of the Consensus document by the World Glaucoma Association on the design and reporting of glaucoma surgical trials [11]. Within each interval, the visit closest to the selected time point was chosen. Data from all centres were combined and queries cross-checked with each surgeon to ensure data accuracy.

### Surgical technique

All patients provided informed consent prior to surgery. The four surgeons used similar surgical techniques with minor differences based on local protocols. All procedures were performed under sub-Tenon’s or subconjunctival anaesthesia. A corneal traction suture was applied, and a fornix-based conjunctival flap created with the surgical plane extended as posteriorly as possible. Minimal cautery was applied to bleeding vessels before application of MMC-saturated corneal sponges underneath the sub-Tenon’s pocket. MMC concentration for all cases was 0.2 mg/ml applied for two minutes. A 1 mm specialised micro-knife was used to create a 2 mm scleral tunnel 3 mm posterior to the marked limbus, and the anterior chamber was penetrated using a 25-gauge needle. The implant was inserted bevel up through the tunnel, and the fins placed just within the sclera. The Tenon’s and conjunctival layers were carefully advanced over the shunt with ample clearance to reduce the risk of postoperative occlusion followed by combined closure of both layers using 9-0/10-0 nylon corner sutures and a mattress suture along the limbus. Postoperatively a standardised regime of topical antibiotic and steroid was prescribed and tapered over two months.

### Definition of success

Definitions of success and failure were chosen in accordance with the World Glaucoma Association consensus guidelines [11]. Four different composite success criteria were used, based on IOP thresholds and percentage IOP reduction from baseline: (1) Criterion-A: IOP ≤ 21 and reduction ≥20%; (2) Criterion-B: IOP ≤ 18 and reduction ≥20%; (3) Criterion-C: IOP ≤ 15 and reduction ≥25%; (4) Criterion-D: IOP ≤ 12 and reduction ≥30%. Success was defined as qualified or complete based on whether this was reached with or without anti-glaucoma medication. Failure was defined when IOP was above predefined criteria at any visit from postoperative month 1, or when one of the following occurred: loss of light perception (LP); hypotony-related complications; inadequate IOP control requiring acetazolamide, surgical revision, or further glaucoma surgery.

The primary outcome was success rates according to the above-mentioned criteria. Secondary outcomes included: IOP, BCVA, medication use, occurrence of complications, postoperative intervention, and factors associated with failure.

### Statistical analysis

Snellen BCVA values were converted to the logMAR scale prior to statistical analysis. Distributions of all variables were visually inspected to assess normality with frequency histograms and quantile-quantile plots. Normal and non-normal continuous variables were reported as mean(±standard deviation) and median(interquartile range) respectively; categorical variables were reported as frequencies or proportions.

Differences in IOP, BCVA, and number of medications at selected time points were tested with linear mixed models, where the patient identification and eye identification were the upper and lower levels of the random effect, respectively, to account for within-subject (two eyes, same patient) and within-eye (same eye repeated measures) correlations. Tukey test was used to test differences between each pair of time points.

Kaplan-Meier survival curves were used to calculate survival probabilities according to the various success criteria and calculate the cumulative incidence of needle revision. We clustered data for patient identification to account for within-subject correlations, and unbiased standard errors were calculated with a robust variance estimate based on the infinitesimal jackknife [12].

Mixed-effects Cox regression models were used to identify preoperative factors associated with failure. All models had a nested effect term with both centre and patient identification as upper and lower levels. As a preliminary step to model fit, a hierarchical cluster analysis was used to explore the degree and patterns of correlation among variables. The following baseline covariates were screened: age, gender, ethnicity (Caucasian vs. non-Caucasian), glaucoma sub-type (primary open angle (POAG) versus other sub-types), lens status, BCVA, IOP, number of topical medications, use of oral acetazolamide, previous laser trabeculoplasty, and previous conjunctival surgery. The least absolute shrinkage and selection operator (LASSO) regression was used to select the variables to enter the final models [13].

Statistical analysis was performed with open-source software R (R Foundation for Statistical Computing, Vienna, Austria). All tests were two-tailed, and *p* values <0.05 were considered statistically significant.

## Results

One hundred eyes of 91 consecutive patients underwent Preserflo™ surgery and were included in this study. All baseline variables had complete observations. Demographic and baseline clinical characteristics are illustrated in Table 1. Overall, the majority of patients were of European descent (95%), had POAG (70%), and were phakic at baseline (69%).

**Table 1:** Demographic and clinical characteristics of patients undergoing Preserflo^TM^ MicroShunt.

No. Eyes/patients	100/91
Age, years, mean (±SD)	67.9 (±12.1)
Ethnicity, *n* (%)	
European descent	86 (95%)
African descent	3 (3%)
Asian descent	2 (2%)
Gender, male/female	52/39
Eye, right/left	53/47
Baseline BCVA, median (IQR)^a^	0.1 (0.0–0.2)
Baseline IOP, median (IQR)	22 (19–28)
Number of glaucoma drops, median (IQR)	3 (2–3)
Acetazolamide, no eyes (%)	20 (20%)
Glaucoma subtype, no eyes (%)	
POAG	70 (70%)
Pseudoexfoliative glaucoma	13 (13%)
Pigmentary glaucoma	7 (7%)
Normal-tension glaucoma	5 (5%)
PACG	3 (2%)
Uveitic glaucoma	2 (2%)
Lens status, no eyes (%)	
Phakic	69 (69%)
Pseudophakic	31 (31%)
Previous LTP, no eyes (%)	15 (15%)
Previous VR Surgery, no eyes (%)	5 (5%)
Previous Glaucoma Surgery (±CEIOL), no eyes (%)	
Trabeculectomy	4 (4%)
Cypass	3 (3%)
Xen Gel Stent	2 (2%)
Viscocanalostomy	2 (2%)
Transscleral Cyclophotocoagulation	3 (3%)
Canaloplasty	1 (1%)
Istent Inject	1 (1%)

*BCVA*  best-corrected visual acuity, *CCT* central corneal thickness, *CEIOL* cataract extraction and intraocular lens implantation, *IQR* interquartile range, *IOP* intraocular pressure, *LTP* laser trabeculoplasty, *NTG* normal-tension glaucoma, *PACG* primary angle-closure glaucoma, *POAG* primary open-angle glaucoma, *SD* Standard deviation; *VR* vitreoretinal.

**Fig. 1:**
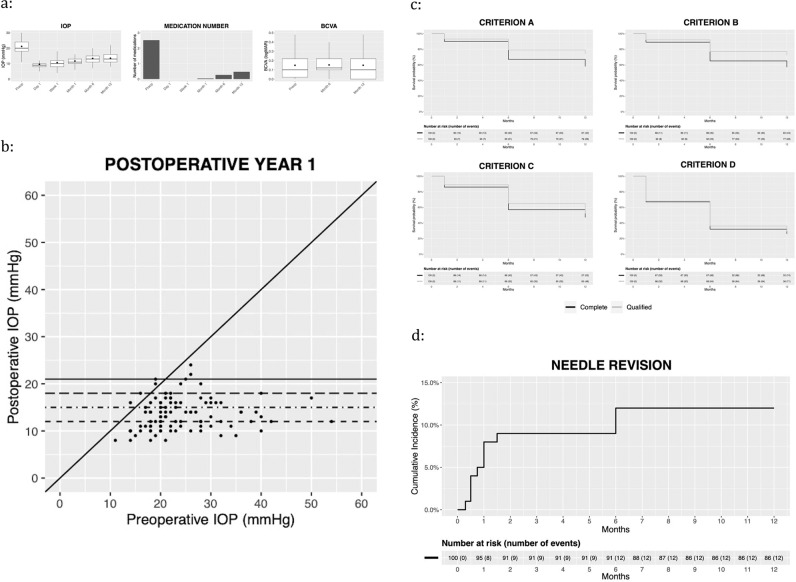
Post-operative outcomes. **a** Intraocular pressure (IOP) readings (left panel), number of medications (middle panel), and best-corrected visual acuity (BCVA) values (right panel) over time in patients undergoing Preserflo MicroShunt. Black diamonds on the boxplots represent mean values. **b** Scatterplot of the preoperative versus postoperative 1-year intraocular pressure values. Dots represent eyes, the diagonal solid line represents the no difference line, and the various horizontal lines indicates the various IOP thresholds (i.e., 21, 18, 15, and 12) used in this study. **c** Kaplan-Meier curves for the qualified (with glaucoma medications) and complete (without medications) success according to the various study criteria. Criterion A: IOP ≤ 21 and IOP reduction ≥20% from baseline; Criterion B: IOP ≤18 and IOP reduction ≥20% from baseline; Criterion C: IOP ≤ 15 and IOP reduction ≥25% from baseline; Criterion D: IOP ≤ 12 and IOP reduction ≥30% from baseline. **d** Kaplan-Meier cumulative incidence for postoperative needling.

Figure 1a illustrates IOP, medication number, and BCVA values over time. Median(IQR) preoperative IOP decreased from 21.5(19–28) mmHg to 13(11–15) mmHg and 13(11–16) mmHg at 6 and 12 months respectively. For every selected time point, postoperative IOP values were always significantly lower than preoperative ones (*p* < 0.001). IOP values tended to be lowest in the first postoperative day, increasing during the first month, and stabilising around month 6. IOP values at day 1, week 1, and month 1 were significantly lower than those measured at months 6 (*p* < 0.001, *p* < 0.001, *p* = 0.043, respectively) and 12 (*p* < 0.001, *p* < 0.001, *p* = 0.028, respectively); IOP values at months 6 and 12 did not significantly differ from each other (*p* = 0.99). As shown in Fig. 1b, postoperative IOP was equal to or greater than preoperative IOP only in a very small number of eyes. The preoperative median (IQR) number of medications decreased from 3(2-3) to 0(0-0) and 0(0-1) at months 6 and 12 respectively. Medication number at any postoperative time point was significantly lower than preoperatively (*p* < 0.001), gradually increasing over time. Preoperative BCVA was not significantly different from postoperative month 6 (median[IQR]: 0.10[0.00–0.22] vs. 0.12[0.07–0.22], *p* = 0.08) and month 12 (median[IQR]: 0.10[0.00–0.22] vs. 0.10[0.00–0.30], *p* = 0.79). Two patients had preoperative BCVA of LP, which was maintained over follow-up. One additional patient had a significant drop in vision to LP due to worsening of pre-existing central retinal vein occlusion with severe macular oedema.

Figure 1c illustrates the Kaplan-Meier success rates as a function of the various success criteria. For Criterion-A, qualified and complete success rates (95%CI) were, 79%(71–87%) and 67%(58–77%) at 6 months, and 74%(66–83%) and 58%(49–69%) at 12 months respectively; for Criterion-B these were 77%(69–86%) and 65%(56–76%) at 6 months, and 72%(63–82%) and 57%(48–68%) at 12 months; for Criterion C, 65%(56–76%) and 57%(47–68%) at 6 months, and 52%(43–63%) and 47%(38–58%) at 12 months respectively and for Criterion D these were 36%(27–47%) and 32%(24–43%) at 6 months, and 29%(21–40%) and 26%(19–36%) at 12 months.

For all qualified success criteria, ethnicity was the only variable selected by LASSO regression for the Cox regression model. As shown in Table 2, non-Caucasian ethnicity was significantly associated with an increased risk of failure for all four criteria. Univariable analysis for all other variables not selected by LASSO regression is shown in Supplementary Table 1; none of these was consistently associated with success.

**Table 2:** Final Cox regression models for factors associated with complete failure according to criteria A, B, C, and D.

Variable	Criteria A Failure		Criteria B Failure		Criteria C Failure		Criteria D Failure	
	*HR (95% CI)*	*P value*	*HR (95% CI)*	*P value*	*HR (95% CI)*	*P value*	*HR (95% CI)*	*P value*
Non-Caucasian ethnicity	8.092 (2.456–26.657)	<0.001	8.348 (2.191–31.804)	0.002	4.479 (1.290–15.552)	0.018	3.206 (1.148–8.954)	0.026

Covariates to enter in the models were selected with LASSO regression.

*CI* confidence interval, *HR* hazard ratio.

Postoperative complications are listed in Table 3a. One patient had device trans-conjunctival erosion four weeks after surgery requiring revision. Two patients with no known previous intraocular inflammation developed anterior uveitis six months post-operatively; both resolved after a short course of topical steroids. One device was implanted too anteriorly causing peripheral corneal oedema requiring removal 8 months post-surgery. The preoperative endothelial cell count for this patient was 1867 cells/mm^2^ and reduced to 800 cells/mm^2^ 8 months later. No corneal surgery was required. In another patient, shunt tip iris incarceration occurred 6 months post-operatively. No hypotony-related complications were observed in our patient cohort.

**Table 3:** **a** Complications after Preserflo^TM^ Microshunt.

Procedures	Eyes (%)
*Early (≤1 month)*	
Device exposure	1 (1%)
*Late (>1 months)*	
Anterior Uveitis	2 (2%)
Peripheral corneal oedema	1 (1%)
Iris incarceration	1 (1%)
Macular oedema secondary to CRVO	1 (1%)
**b**. Other procedures after Preserflo^TM^ MicroShunt	
Needle revision	12 (12%)
5-FU deposit	5 (5%)
Surgical revision for encapsulation	4 (4%)
Surgical revision for exposure	1 (1%)
Device removal	1 (1%)

*CRVO* central retinal vein occlusion, *5-FU* 5-fluorouracile.

Overall, 12(12%) eyes underwent one or more needlings in the first year with the cumulative incidence being 8.0%(1.9–3.7%), 9.0%(2.6–15%), and 12%(4.8–18.6%) at 1, 4, and 12 months, respectively, Fig. 1d. All other post-operative procedures are listed in Table 3b.

## Discussion

In this multicentre study, we retrospectively evaluated the 1-year success rates and safety profile of Preserflo™, a new less invasive glaucoma surgery. Overall, the device provided a significant reduction in IOP values and medications number. The success rates of the procedure varied according to the IOP cutoff chosen. Preserflo™ achieved good success rates for the higher IOP cutoffs, with almost three-quarters of patients maintaining sustained IOPs of 18 mmHg or less with ≥20% reduction, two-thirds of which were off all topical treatment. On the other hand, success rates for the most stringent criterion (IOP ≤ 12 mmHg with ≥30% IOP reduction) were modest, with less than one-third being regarded successful, irrespective of drop use. The device proved to be very safe overall, with no hypotony-related complications occurring in our patient cohort, and provided smooth post-surgical care with few manipulations, particularly in the early postoperative period.

Surgical management of glaucoma varies widely amongst clinicians worldwide. Trabeculectomy, classically considered the gold standard of glaucoma surgery, is now being challenged by newer procedures. Our study provides “real-life” success rates for Preserflo™, with highly generalisable results because of the unrestrictive study criteria and the multicentre/multinational design. Direct comparisons of our results with those of other studies using Preserflo™ or other surgical techniques are difficult due to differences in methodology, demographics, surgical approaches, reported outcomes, and degree of loss to follow-up.

Schlenker and colleagues [7] have recently reported one-year Preserflo™ results in a large patient cohort (164 eyes) from a single tertiary referral centre. They reported a median IOP reduction from 20 mmHg to 12 mmHg, which is similar to our study (21.5–13 mmHg). For all IOP cut-offs, success was higher than in our study with, for example, 93% versus 77% for criteria comparable to criterion B. One possible explanation for this difference is that higher MMC concentrations were used in most patients, with this being associated with increased survival rates within the study itself. Their analysis also did not include patients at high risk of failure, with exclusion criteria including more aggressive glaucoma sub-types and previous intraocular surgery. Furthermore, all patients were treated by a single, world-renowned, glaucoma surgeon, thereby results might not be generalisable. Our results involved the very first 25 cases of four experienced glaucoma surgeons, irrespective of glaucoma sub-type or previous surgical history, and using a fixed dose of MMC. Subsequent to these first cases, all four surgeons have switched to higher MMC doses, which would presumably change success outcomes.

Baker et al. have just recently published one-year interim results of a large, ongoing, 2-year, randomised, multicentre study comparing Preserflo™ directly with trabeculectomy in POAG [9]. In 395 patients randomised to Preserflo™, the reported success rate was 53.9% (versus 72.7% in trabeculectomy) for a 20% reduction in baseline IOP, with a mean IOP drop from 21.1 to 14.3 mmHg. Compared to this data, our patients had a larger IOP reduction and significantly higher success rates, the latter even compared with the trabeculectomy group, despite the more lax success criterion (20% IOP reduction alone). Interestingly, Baker’s group used a fixed lower dose of MMC (0.2 mg/ml), as in our own study. They also reported a 40.8% incidence of post-operative interventions and 28.9% transient hypotony; our study showed significantly lower rates of the former (23%) whilst no significant ocular hypotony was reported. The latter however might be a reflection of post-operative follow-up plans: all patients were day cases and reviewed 5–7 days post-surgery, potentially missing transient low IOP in the early days.

The Primary Tube Versus Trabeculectomy (PTVT) study [14] reported mean IOP reductions from 23.3 mmHg to 13.8 mmHg and 23.9 mmHg to 12.4 mmHg in surgically naïve glaucoma patients receiving augmented trabeculectomy or Baerveldt tube implantation implant respectively. Our results fair similarly to the tube arm and are marginally higher than the trabeculectomy arm. In addition, the 1-year success rates of trabeculectomy and tubes were, respectively, 92 and 83% for IOP ≤ 21 mmHg, 90 and 79% for IOP < 18 mmHg, and 80 and 72% for IOP < 15 mmHg. Our success rates are considerably lower than those reported in the PTVT, with this difference becoming more obvious as IOP cut-offs decrease. Reasons for this discrepancy may include the fact that Preserflo™ is less invasive, and therefore not expected to achieve such low sustained IOP control. The inclusion of high-risk eyes, learning curve, and more stringent success criteria may also explain these differences. Compared to both PTVT groups however, Preserflo™ had a much lower complication rate and required less intensive postoperative management. For example, approximately two-thirds of patients in either PTVT arm required a postoperative intervention at one year. In comparison, only around one-quarter of our patients required postoperative manipulations. At 1 year, the complication rate for trabeculectomy and tubes were, respectively, 33% and 20% for early (onset ≤ 1 month) and 15% and 16% for late (onset > 1 month) complications. This compares to our study, which had early complication rate of 1% and late complication rate of 5%. As our study was retrospective with data abstracted from clinical charts, complications, especially minor ones, could have been under-reported. Still, our overall complication rates were considerably lower than rates reported by the PTVT study. No hypotony-related complication occurred in our cohort of patients, as opposed to 10% and 8% for choroidal effusion and 5% and 1% for hypotony maculopathy reported at 1 year by the PTVT investigators in the trabeculectomy and tubes arms, respectively. Our study confirms that Preserflo™ Microshunt is a very safe surgical technique, especially when it comes to avoiding hypotony complications.

Other “less invasive” sub-conjunctival drainage devices have become popular over the last decade. Reitsamer et al. analysed the results of Xen-45™ in a prospective multicentre study, reporting a mean IOP reduction from 21.4 mmHg to 14.9 mmHg at 12 months [15]. In a retrospective study, Scheres and colleagues found similar results, with Xen-45™ producing a mean IOP reduction from 18.4 mmHg to 14.4 mmHg at 12 months, and 1-year qualified success rates of 78% for IOP ≤ 18mmHg [16]. The success rates and IOP reduction provided by Preserflo™ in our study seem to be at least as good as those reported for Xen-45™. However, despite similar efficacy, Xen-45™ requires more intensive postoperative management, with up to 41% of patients subsequently undergoing needling procedures [15, 16]. Additionally, some surgeons feel that needling Xen-45™ blebs is technically challenging and can even cause implant damage [17]. In our cohort, only 12% of patients required bleb needling, resulting in smoother postoperative management.

In this study, we performed an explorative analysis to identify factors associated with increased risk of failure according to various IOP criteria. This showed that patients of Non-Caucasian ethnicity had a higher rate of failure compared to those of Caucasian ethnicity for all criteria. This is not surprising as many studies have shown that patients of European descent have less aggressive postoperative scarring and better outcomes. Our analysis was limited by the small case numbers of non-Caucasian patients and the exact magnitude of ethnicity impact is uncertain as confidence intervals were very large, with different non-Caucasian ethnicities pooled into a single category. Further studies with more diverse ethnic populations are required to confirm and further characterise this finding. No other variables appeared to affect success or failure of this procedure. Schlenker et al. [7] also evaluated these factors and did not find any impact of ethnicity; they did however find that low MMC dosage (0.2 mg/mL), as used in our study, was associated with increased failure compared to a higher dosage (0.4 mg/mL). Future studies with longer follow-up and heterogeneity in MMC concentration will hopefully clarify the impact of MMC dosage on long-term efficacy and safety of Preserflo™ surgery.

Our results were based on the first 25 cases performed by surgeons who had extensive prior experience of both trabeculectomy and glaucoma drainage devices (tubes). Overall, the learning curve was short and safe, with intraoperative complications occurring in only one patient. Other surgeries aiming to minimise trabeculectomy complications have been previously developed, including ‘non-penetrating glaucoma surgeries’ such as deep sclerectomy and canaloplasty. Although these procedures have favourable long-term safety and efficacy profiles, they have not been widely adopted for reasons including their technical complexity, steep learning curve, and high incidence of intraoperative complications during the learning phase [18, 19].

This study has several limitations. Firstly, because of its retrospective nature, study time points were not set in advance but were artificially defined to standardise the unequal visit intervals across centres. The number of time points was small, therefore limiting chances to detect failure. Also, the determined time of failure may not be accurate; since time points were widely spaced in time, this could have occurred at any point in between consecutive visits. Secondly, this study is non-comparative and does not provide information on how Preserflo™ compares with other established glaucoma surgeries. The main outcome measure was IOP control, which is a surrogate and imperfect measure for structural and functional success. Although Preserflo™ proved to be effective in the short-term, the study does not provide information about long-term outcomes. Some variables of interest, such as cornea endothelial count, were not available as specular microscopy is not routinely performed by all but one of the surgeons involved in this study. Because of the small sample size and limited number of patients in some subsets (e.g., eyes with non-virgin conjunctiva), some analysis could be underpowered. For all Cox analyses, we have reported 95% CIs along with HRs and *p* values. We invite the reader to evaluate HRs and *p* values in associations with 95% CIs to understand whether a certain analysis is underpowered. Nonsignificant predictors with wide 95% CI, which spans loosely around a HR of 1 may indicate a type II error (i.e., analysis underpowered to detect a true association); on the other hand, significant variables with wide CIs may indicate a true association, whose magnitude is uncertain.

In conclusion, Preserflo™ is a viable surgical option in glaucoma patients, with reasonable short-term success rates, decreased medications use, excellent safety profile, smooth postoperative care, and rapid learning curve. Success rates for the most stringent IOP cutoffs were modest, indicating that this device may not be the optimal surgery in patients requiring a low target IOP, such as those with advanced glaucoma or low pre-operative pressures. Further studies are required to clarify the long-term success rates in comparison with established glaucoma surgical techniques and to identify the best candidates for this procedure.

Supplemental information is available at Eye’s website.

### Summary

#### What was known before

- Less invasive glaucoma surgeries (LIGS) are becoming more popular in treatment algorithms of progressing glaucoma patients.
- The use of LIGS is increasing significantly compared to traditional trabeculectomy due to the perceived risk of hypotony, intensive postoperative management and significant learning curve.

#### What this study adds

- Preserflo Microshunt is a viable surgical option in glaucoma patients, with reasonable short-term success rates, decreased medications use, excellent safety profile, smooth postoperative care, and rapid learning curve.

## Supplementary information

Supplemental Table 1

## References

[1] Chen DZ, Sng CCA (2017). Safety and efficacy of microinvasive glaucoma surgery. J Ophthalmol.

[2] Conlon R, Saheb H (2017). Ahmed, II. Glaucoma treatment trends: a review. Can J Ophthalmol.

[3] Holland LJ, Mercieca KJ, Kirwan JF. Effect of COVID-19 pandemic on glaucoma surgical practices in the UK. *Br J Ophthalmol.* 2021. 10.1136/bjophthalmol-2021-319062 33931388

[4] Acosta AC, Espana EM, Yamamoto H, Davis S, Pinchuk L, Weber BA (2006). A newly designed glaucoma drainage implant made of poly(styrene-b-isobutylene-b-styrene): biocompatibility and function in normal rabbit eyes. Arch Ophthalmol.

[5] Batlle JF, Fantes F, Riss I, Pinchuk L, Alburquerque R, Kato YP (2016). Three-year follow-up of a novel aqueous humor MicroShunt. J Glaucoma.

[6] Pinchuk L, Riss I, Batlle JF, Kato YP, Martin JB, Arrieta E (2016). The use of poly(styrene-block-isobutylene-block-styrene) as a microshunt to treat glaucoma. Regen Biomater.

[7] Schlenker MB, Durr GM, Michaelov E, Ahmed IIK (2020). Intermediate outcomes of a novel standalone ab externo SIBS microshunt with mitomycin C. Am J Ophthalmol.

[8] Durr GM, Schlenker MB, Samet S, Ahmed IIK. One-year outcomes of stand-alone ab externo SIBS microshunt implantation in refractory glaucoma. *Br J Ophthalmol*. 2020;106:71–9. 10.1136/bjophthalmol-2020-317299 33097520

[9] Baker ND, Barnebey HS, Moster MR, Stiles MC, Vold SD, Khatana AK, et al. Ab-externo MicroShunt versus trabeculectomy in primary open-angle glaucoma: 1-year results from a 2-year randomized, multicenter study. *Ophthalmology.* 2021;128:1710–21. 10.1016/j.ophtha.2021.05.023 34051211

[10] Beckers HJM, Aptel F, Webers CAB, Bluwol E, Martinez-de-la-Casa JM, Garcia-Feijoo J, et al. Safety and effectiveness of the PRESERFLO(R) MicroShunt in primary open-angle glaucoma: results from a 2-year multicenter study. *Ophthalmol Glaucoma.* 2021;28;179–4. 10.1016/j.ogla.2021.07.008 34329772

[11] Shaarawy TM, Sherwood MB, Grehn F. *Guidelines on design and reporting of glaucoma surgical trials*. Kugler Publications; 2009.

[12] Therneau TM, Grambsch PM. *Modeling survival data: extending the Cox model*. Springer-Verlag New York: New York; 2000.

[13] Tibshirani R (1997). The Lasso method for variable selection in the Cox model. Stat Med.

[14] Gedde SJ, Feuer WJ, Shi W, Lim KS, Barton K, Goyal S (2018). Treatment outcomes in the primary tube versus trabeculectomy study after 1 year of follow-up. Ophthalmology.

[15] Reitsamer H, Sng C, Vera V, Lenzhofer M, Barton K, Stalmans I (2019). Two-year results of a multicenter study of the ab interno gelatin implant in medically uncontrolled primary open-angle glaucoma. Graefes Arch Clin Exp Ophthalmol.

[16] Scheres LMJ, Kujovic-Aleksov S, Ramdas WD, de Crom R, Roelofs LCG, Berendschot T, et al. XEN((R)) Gel Stent compared to PRESERFLO MicroShunt implantation for primary open-angle glaucoma: two-year results. *Acta Ophthalmol*. 2020;99:e433–40. 10.1111/aos.14602 PMC8246811 32909682

[17] Olivari S, Cutolo CA, Negri L, Cappelli F, Testa V, Iester M (2019). XEN implant fracture during needling procedure. J Glaucoma.

[18] Rabiolo A, Leadbetter D, Alaghband P, Anand N (2021). Primary deep sclerectomy in open-angle glaucoma: long-term outcomes and risk factors for failure. Ophthalmol Glaucoma.

[19] Mendrinos E, Mermoud A, Shaarawy T (2008). Nonpenetrating glaucoma surgery. Surv Ophthalmol.

