# Supplementary material for: Short-term safety and efficacy of Preserflo™ Microshunt in glaucoma patients: a multicentre retrospective cohort study
Source: Eye (Lond). 2022 Mar 12;37(4):644–9. doi: 10.1038/s41433-022-01995-7 (PMC8916945; doi:10.1038/s41433-022-01995-7)
Supplement: Supplementary file 1 — Supplemental Table 1 [file 41433_2022_1995_MOESM1_ESM.docx]

| **Supplementary Table 1.** Univariable Cox Regression Models for factors not selected by LASSO regression for complete failure according to criteria A, B, C, and D. | | | | | | | | |
| --- | --- | --- | --- | --- | --- | --- | --- | --- |
|  | **Criteria A Failure** | | **Criteria B Failure** | | **Criteria C Failure** | | **Criteria D Failure** | |
| **Variable** | *HR*  *(95% CI)* | *P-value* | *HR*  *(95% CI)* | *P-value* | *HR*  *(95% CI)* | *P-value* | *HR*  *(95% CI)* | *P-value* |
| Age, years | 0.987  (0.961-1.014) | 0.33 | 0.989  (0.960-1.019) | 0.46 | 0.994  (0.968-1.021) | 0.66 | 1.001  (0.982-1.022) | 0.89 |
| Male gender | 1.453  (0.741-2.850) | 0.28 | 1.344  (0.649-2.784) | 0.43 | 2.007  (1.037-3.887) | **0.039** | 1.480  (0.898-2.438) | 0.12 |
| Other Glaucoma subtypes  (ref:POAG) | 1.072  (0.497-2.315) | 0.86 | 0.905  (0.398-2.056) | 0.81 | 0.898  (0.427-1.892) | 0.78 | 0.734  (0.421-1.280) | 0.28 |
| Baseline Pseudophakia | 0.645  (0.301-1.383) | 0.26 | 0.762  (0.340-1.708) | 0.51 | 0.840  (0.412-1.712) | 0.63 | 0.916  (0.485-1.731) | 0.79 |
| Previous LTP | 0.764  (0.284-2.054) | 0.59 | 0.755  (0.259-2.200) | 0.61 | 0.963  (0.382-2.426) | 0.94 | 0.787  (0.367-1.685) | 0.54 |
| Previous Conjunctival Surgery | 0.895  (0.367-2.184) | 0.81 | 0.836  (0.313-2.238) | 0.72 | 0.948  (0.401-2.242) | 0.90 | 0.897  (0.441-1.825) | 0.76 |
| Preoperative BCVA, logMAR | 1.584  (0.812-3.091) | 0.18 | 1.496  (0.721-3.104) | 0.28 | 1.307 (0.651-2.624) | 0.45 | 1.441  (0.822-2.528) | 0.20 |
| Preoperative IOP, mmHg | 0.960  (0.913-1.009) | 0.11 | 0.967  (0.919-1.018) | 0.20 | 0.998  (0.957-1.040) | 0.92 | 1.021  (0.991-1.053) | 0.18 |
| Preoperative meds, n | 0.916  (0.625-1.340) | 0.65 | 0.933  (0.618-1.407) | 0.74 | 0.744  (0.512-1.081) | 0.12 | 1.031  (0.770-1.381) | 0.84 |
| Preoperative Acetazolamide | 0.781  (0.329-1.853) | 0.58 | 0.733  (0.287-1.869) | 0.52 | 0.859  (0.375-1.967) | 0.72 | 0.916  (0.485-1.731) | 0.79 |
| For continous variables, HR are intended for each 1-unit increase.  BCVA: best corrected visual acuity; CI: confidence interval; HR: hazard ratio; IOP: intraocular pressure; LTP: laser trabeculoplasty; POAG: primary open-angle glaucoma. | | | | | | | | |
